# *Brucella ceti* in Common Dolphins (*Delphinus delphis*) in Portugal—Characterization of First Isolates

**DOI:** 10.3390/ani15030374

**Published:** 2025-01-28

**Authors:** Sandra Cavaco, Miguel L. Grilo, Ricardo Dias, Mónica Nunes, Pedro Pascoal, Marcelo Pereira, Catarina Fogaça, Ana Beatriz Costa, Sofia Pardal, Ana Cristina Ferreira

**Affiliations:** 1 National Institute for Agrarian and Veterinary Research (INIAV IP), Av. da República, Quinta do Marquês, 2780-157 Oeiras, Portugal; sandra.cavaco@iniav.pt; 2 RALVT—Rede de Arrojamentos de Lisboa e Vale do Tejo, ISPA—Instituto Universitário de Ciências Psicológicas, Sociais e da Vida, Rua Jardim do Tabaco 34, 1149-041 Lisbon, Portugal; mgrilo@ralvt.pt (M.L.G.); catarinafogacavet@gmail.com (C.F.); anacostafc98@gmail.com (A.B.C.); spardal@ralvt.pt (S.P.); 3 Egas Moniz Center for Interdisciplinary Research (CiiEM), Egas Moniz School of Health & Science, Campus Universitário, Quinta da Granja, 2829-511 Almada, Portugal; 4 MARE—Marine and Environmental Sciences Centre/ARNET—Aquatic Research Network, ISPA—Instituto Universitário de Ciências Psicológicas, Sociais e da Vida, Rua Jardim do Tabaco 34, 1149-041 Lisbon, Portugal; 5 cE3c—Centre for Ecology, Evolution and Environmental Changes & CHANGE—Global Change and Sustainability Institute, Faculdade de Ciências, Universidade de Lisboa, Campo Grande, 1749-016 Lisbon, Portugal; rpdias@fc.ul.pt (R.D.); msnunes@ciencias.ulisboa.pt (M.N.); pfpascoal@fc.ul.pt (P.P.); 6 BioISI-Biosystems and Integrative Sciences Institute, Faculty of Sciences, University of Lisbon, 1749-016 Lisbon, Portugal; mlpereira@fc.ul.pt; 7 I-MVET-Faculty of Veterinary Medicine, Lusófona University, University Centre of Lisbon, 1749-024 Lisbon, Portugal

**Keywords:** Atlantic Sea, *Brucella ceti*, cetaceans, comparative genomics, infection surveillance, marine environment health, Portugal

## Abstract

This study investigates *Brucella ceti* infection in marine mammals stranded along the Lisbon and Tagus Valley coast between 2022 and mid-2024, marking the first report of *Brucella* presence in Portuguese waters. Out of 59 examined marine mammals, *B. ceti* was isolated in three common dolphins (5.1%), a prevalence rate consistent with previous studies from other coastlines. PCR-based detection indicated a higher infection rate (23.7%), suggesting an underestimation of the prevalence of *B. ceti* infection in this population. Multi-locus Sequence Typing (MLST) and Multiple-Locus Variable-Number Tandem-Repeat Analysis (MLVA) revealed distinct genetic profiles and close relationships to *B. ceti* strains from the Atlantic, supporting the hypothesis of specific host-adapted lineages in dolphins. Virulence genes, including those for host interaction (*bsp*E, *btp*B) and intracellular survival (*vir*B7, *vce*A), were consistent across isolates, highlighting the pathogenic potential. Additionally, antimicrobial resistance (AMR) genes, such as *mpr*F and efflux proteins (*bep*C-G), were also identified. These findings underscore the need for further research and surveillance to understand *B. ceti* transmission, host range, and impacts on Atlantic cetaceans, as well as to develop effective diagnostic and management strategies to mitigate infection risks in marine environments.

## Simple Summary

This study investigates *Brucella ceti* infections in marine mammals stranded along the Lisbon and Tagus Valley coast from 2022 to mid-2024, reporting the first evidence of this zoonotic agent in Portuguese waters. Among 59 animals, *B. ceti* was isolated in 5.1% of dolphins, with a higher PCR-based detection rate (23.7%), suggesting an underestimation of infection prevalence. Genetic analysis revealed links to Atlantic strains, supporting host-adapted lineages in dolphins. Key findings include virulence genes (*bsp*E, *btp*B, *vir*B7, *vce*A) and antimicrobial resistance genes (*mpr*F, *bep*C-G), highlighting pathogenic potential. The findings emphasize the need for further research and surveillance to understand and manage infection risks.

## 1. Introduction

*Brucella* infections of terrestrial mammals have long been recognized and extensively researched; however, it was only during the last years of the twentieth century that the first reports on *Brucella* species from animals living in the marine environment were made, leading later to the inclusion of two novel species in the genus: *Brucella ceti* and *Brucella pinnipedialis* [1]. The first reports on *Brucella* sp. isolates from marine mammals were from wild harbor seals (*Phoca vitulina*), a harbor porpoise (*Phocoena phocoena*), and a common dolphin (*Delphinus delphis*) in Scotland [2], and a captive bottlenose dolphin (*Tursiops truncatus*) in the USA [3]. *B. ceti* infections have been frequently described in different dolphin species from the Atlantic and the Pacific Oceans [4], as well as from the Mediterranean Sea [5], and they have been recognized as a significant health concern for cetacean populations [4]. These reports have significantly broadened the range of host species known to be affected. In addition, they have extended the area over which the infection is known to occur, to the point where, if serological evidence is included, it seems likely that *Brucella* infection among sea mammals has a global occurrence [4].

*B. ceti* and *B. pinnipedialis* have been isolated from lungworms and a variety of organs in marine mammals [1,4,5,6,7,8,9,10,11,12]. In dolphins, *B. ceti* infections can be a challenging diagnosis, as clinical signs are nonspecific and depend on the affected organ or system. This infection is usually associated with meningoencephalomyelitis [1,3,4,7,8,9,10], reproductive tract inflammation (orchitis, endometritis, placentitis, endometritis), mastitis, abortion [1,2], discospondylitis, subcutaneous abscesses, and a wide range of other pathological conditions like pneumonia, myocarditis, pericarditis, osteoarthritis, hepatic, splenic, and lymph node necrosis, alongside macrophage infiltration in the liver and spleen, [1,2,3,4,5,6,7,8,9]. Neurobrucellosis represents a common cause of stranding for cetaceans, being associated with disorientation, uncoordinated lateral swimming, buoyancy disturbances, and death [4,5,9,10].

Grattarola and colleagues [10], among different bacteria with a potential zoonotic role identified in cetaceans stranded along the Italian Coastline, found that *Brucella* spp. were one of the most represented bacterial species, with a prevalence of 4.92%. In fact, there are several reports on *B. ceti* isolation on Italian and Spanish Mediterranean coasts from striped and bottlenose dolphins [8,9,10,11,12]. Additionally, a recent review from Jamil and Colleagues [13] highlights the presence of *B. ceti* and *B. pinnipedialis* infections in different marine mammal species across European regions, including the North Atlantic Ocean, the Russian Bering Island, the Croatian Adriatic Sea, the Netherlands, Germany, and Norway. These epidemiological studies are crucial in elucidating the transmission dynamics, prevalence, and risk factors associated with *Brucella* spp. infection in marine mammal populations, not only offering valuable insights into the prevalence and distribution of the pathogen but also informing targeted intervention strategies to help mitigate its spread. Although there are no studies on the prevalence of *Brucella* in wild marine mammals in Portugal, the results above described in surrounding areas, lead us to hypothesize that the populations of marine mammals that arrive and pass through Portugal may also bear this bacterial infection.

Identifying *B. ceti* as the causative agent of disease and conducting comprehensive risk assessments are fundamental steps in developing effective biosafety protocols. By characterizing the virulence factors, antibiotic resistance profiles, and transmission routes of *B. ceti*, researchers can assess the potential risks posed to marine mammal populations, aquaculture facilities, and Public Health. *B. ceti* infections in marine mammals can pose a concern for marine conservation efforts [4], as increased pathogen prevalence might interfere with population abundance, by inducing high mortality rates, lowering reproductive success, or by synergistically increasing the pathogenicity of other diseases.

In this study, *Brucella* infection in stranded marine mammals in the region of Lisbon and Tagus Valley (Portugal) was investigated to determine prevalence rates, species identification, phylogenetic relationships, and comparative genomic analysis. This is the first time *B. ceti* has been isolated from dolphins from the Portuguese coastline.

## 2. Materials and Methods

### 2.1. Stranding Data Collection, Necropsy, and Tissue Sampling

**Figure 1:**
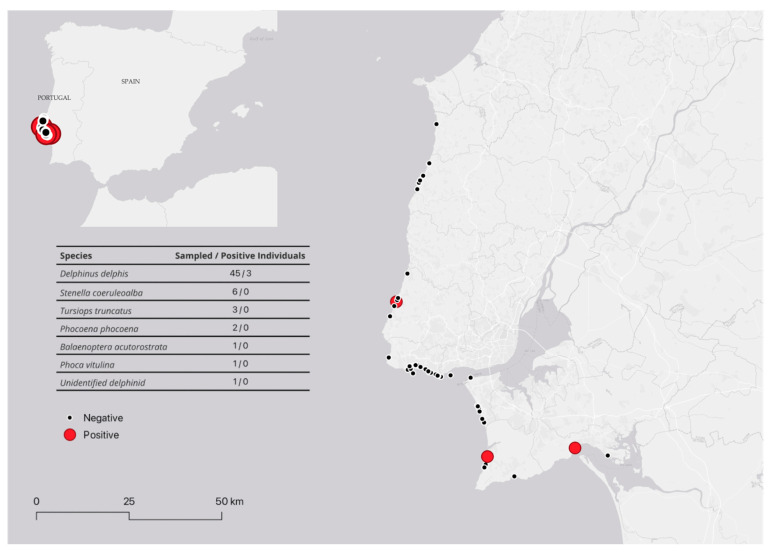
Geographical distribution of the stranding events and species under study.

Post-mortem examinations were performed on 59 marine mammals (*Balaenoptera acutorostrata, n* = 1; *Delphinus delphis, n* = 45; *Phoca vitulina, n* = 1; *Phocoena phocoena, n* = 2; *Stenella coeruleoalba, n* = 6; *Tursiops truncatus, n* = 3; Unidentified, *n* = 1) stranded in different beaches along the Lisbon and Tagus Valley coastline, specifically between Lourinhã and Setúbal municipalities, between January 2022 and June 2024. Due to logistical and resource constraints, post-mortem investigations in this study were limited to gross pathological findings, as histopathological analysis was not performed. Geographical distribution of the stranding events and species under study is shown in Figure 1. Necropsy and macroscopic evaluations, as well as sample collection, were performed by the Lisbon and Tagus Valley Marine Animals’ Stranding Network. Timelapse between receiving the stranding alert and performing necropsy and sample collection was under 12 h in all the investigated animals. A detailed post-mortem examination was performed according to standard protocols [14], and sample collection depended on the carcasses’ preservation status. Individual data, including sex, decomposition code, and nutritional condition, along with stranding data concerning the geographical location, and date of occurrence, were registered. During necropsies, samples from tissues (brain, spleen, liver, lung, mammary gland, testis/uterus, and several lymph nodes), vaginal and preputial swabs, and/or fluids (blood, milk) were collected, and kept frozen at <−20 °C until tested. All samples (Supplementary File Table S1) were submitted to the Animal Health National Reference Laboratory (INIAV, Oeiras, Portugal) for microbiological and molecular investigations focused on *Brucella* infection diagnosis.

### 2.2. Brucella Isolation and Identification

The primary isolation of *Brucella* spp. was performed on all tissues, swabs, and fluids available from the 59 animals stranded. Samples were homogenized under sterile conditions in the minimum possible amount of sterile buffered saline (PBS pH 6.8) in a Stomacher unit (Seward Medical, Worthing, UK), and 0.2 mL of each tissue homogenate was inoculated on two plates of both Farrell’s [15] and CITA [16] selective media. The plates were observed for microbial growth following 5–10 days of incubation at 37 °C in both ambient air and a 5% CO₂ environment. A culture was considered positive when at least one *Brucella* colony-forming unit (CFU) was isolated. Suspected colonies were further identified and characterized by standard bacteriological procedures, based on CO_2_ requirement, H_2_S production, oxidase test, urea hydrolysis, agglutination with monospecific sera anti-A, anti-M, anti-R, and fuchsin and thionine dye sensitivity [17].

### 2.3. Molecular Methods

Bacterial genomic DNA extraction from available tissues was performed in a nucleic acid extraction workstation, Kingfisher Flex (Thermo Fisher Scientific, Waltham, MA, USA), using the IndiMag Kit (Indical Bioscience, Leipzig, Germany), following the manufacturer’s instructions. After extraction, the DNA was stored at 4 °C. All samples were tested using RT-PCR targeting *bcsp*31 and *per* genes, as described previously [18], for identification of *Brucella* spp. Briefly, real-time TaqMan PCR was set up in a final volume of 25 µL, 1× TaqMan Universal PCR Master Mix (Applied Biosystems, Foster City, CA, USA), each primer and TaqMan probe at concentrations of 0.3 µM and 0.25 µM, respectively, and 3 ng of DNA template. The reaction mixture was initially incubated for 10 min at 95 °C. Amplification was performed for 45 cycles of denaturation at 95 °C for 15 s, annealing, and extension at 60 °C for 1 min. The PCR reaction was performed on a Bio-Rad CFX Maestro 2.3 (Bio-Rad, Hercules, CA, USA). A result was considered positive when an amplification curve with a Ct value less than 38 was obtained for both targets.

Genomic DNA from each *Brucella* spp. isolated from this work, and the control strains, was extracted with PureLink Genomic DNA Mini Kit (Invitrogen, MA, USA), and stored at −20 °C until used. Brucellae isolates were identified using the multiplex PCR Bruce-ladder as described elsewhere [19]. DNA samples were also tested by Multiple-loci variable number of tandem repeats analysis (MLVA-16), as previously described [20]. The 16 *loci* have been classified in three panels, named panel 1, composed of 8 minisatellite (bruce06, bruce08, bruce11, bruce12, bruce42, bruce43, bruce45, and bruce55), panel 2A (bruce18, bruce19, and bruce21), and panel 2B (bruce04, bruce07, bruce09, bruce16, and bruce30), and composed of three and five microsatellite markers, respectively. Briefly, PCR reactions were performed in a total volume of 15 μL containing 3 ng of DNA, 1 × PCR Reaction Buffer, 1 U of Taq DNA polymerase (Promega, MI, USA), 200 μM of each dNTPs, and 0.3 μM of each flanking primers. An initial denaturation step at 96 °C for 5 min was followed by 30 cycles of denaturation at 96 °C for 30 s, primer annealing at 60 °C for 30 s and elongation at 70 °C for 1 min. The final extension step was performed at 70 °C for 5 min. Amplification products were loaded on a 3% standard agarose gel to analyze panel 2A and 2B *loci* (tandem repeats with a unit length shorter than 8 bp), and on a 2% standard agarose gel for panel 1 *loci* (tandem repeats with a unit length larger than 10 bp), with suitable molecular size markers. The total number of repeats at each locus was determined by the correlation with the amplicon size according to the 2013 *Brucella* allele assignment table (Le Flèche et al., 2006 version 3.6 available at https://microbesgenotyping.i2bc.paris-saclay.fr/databases/view/61 accessed on 27 January 2025). Genomic DNA from *B. melitensis* biovar 1 strain 16 M (ATCC 23456) and *B. ceti* Atlantic dolphin type (B14/94) were used as controls for allele assignment.

### 2.4. Whole-Genome Sequencing and Bioinformatic Analysis

Whole-Genome Sequencing (WGS) was used to evaluate the genetic structure of *B. ceti* isolates to increase knowledge of brucellae genome evolution. The extracted nucleic acids were assessed for quality and quantity using spectrophotometry and dsDNA-specific fluorescence-based assays. WGS was performed as previously described [21,22]. Briefly, to recover the genomic structure of these isolates as accurately as possible, the extracted long-strand high-grade DNA was directly sequenced using native long-read Nanopore sequencing (GridION X5 sequencing platform, Oxford Nanopore Technologies, Oxford, UK) at a minimum coverage depth of 200×, using Nanopore V14 kit chemistry with R10.4.1 pore. The sequencing data were analyzed with a customized pipeline developed by BioISI Genomics for genome assembly. The analysis included base-calling, pre-filtering based on read size and quality, and subsequent assembly, which involved the taxonomical classification using Kraken version 2.1.2 [23], Database NCBI nt 2023, and plasmid identification with PlasmidFinder [24]. Phylogenetic reconstruction was performed using ParSNP [25], with subsequent visualization of phylogenetic trees and graphical representation of mutations generated using Gingr version 1.3 [26], where purple lines represent identified mutations, and gray bands indicate regions without alignment. The variant call output from ParSNP was further annotated using Prokka version 1.14.6 [27], for identification of the specific mutations of sample DDE001 and of the group DDE002 and DDE003. Pathogenicity prediction was determined using PathogenFinder (v1.1.) [28], identification of acquired virulence genes via VirulenceFinder (v2.0) and Virulence Factor Database—VFDB (v6.0) [29], identification of antibiotic resistance genes with CARD (v3.3.0) [30], MegaRES (v3.0) [31], and ResFinder (v4.5.0) [32]. Additionally, Multi-Locus Sequence Typing (MLST) [33,34] was performed using the PubMLST database (v2.0, accessed on 21 March 2024), and Multiple-Locus Variable-number Tandem Repeat Analysis (MLVA) which followed the MLVA-16 scheme described by Maquart et al. [35], with data obtained from the MLVA bank for microbes genotyping (v1.4.0, accessed on 21 March 2024).

For bioinformatic analysis, only reads with a Q score > 7 (minimum value from MinKNOW) were retained. Duplex base calling was performed using Dorado (v0.3.1+bb8c5ee) with the dna_r10.4.1_e8.2_400bps_sup@v4.2.0 model. Assembly was conducted with Canu (v2.0) [36] using parameters: genomeSize = 3.4M and -nanopore-raw. A quality report for the assemblies was generated using Quast (v5.0.2) with parameters set for a prokaryotic genome assembly. The Canu assemblies served as input for various bioinformatic tools available at https://genomicepidemiology.org/services/ (accessed on 27 January 2025): Abricate v1.0.1 with VFDB [28,37], CARD, MegaRES [30,31], and PlasmidFinder (v2.1, “Gram Negative” database option) [24]. The assemblies were also analyzed using MLST tool (v2.0, with the configuration specific to *Brucella* spp.) and MLVA bank for Microbes genotyping (v1.4.0, configured for *Brucella* v4_6_5).

## 3. Results

### 3.1. Brucella Infection Diagnosis and Brucella Isolates Characterization

The isolation of *Brucella* spp. suspect colonies was obtained in three common dolphins (*Delphinus delphis*) out of the 59 animals investigated (5.1%). Suspected colonies were isolated from brain in DDE001 and DDE003 dolphins, and from spleen, liver, lung, and lymph nodes in all three animals. All isolates were typed by bacteriological and molecular methods and assigned as *B. ceti*. The phenotypic features regarding CO_2_ requirement, H_2_S production, oxidase and urea tests, agglutination with monospecific sera, and fuchsin and thionine sensitivity were consistent with previously described *B. ceti* patterns: none of the three isolates required CO_2_ for growth or produced H_2_S, but all presented smooth phenotype, were oxidase- and urease-positive, agglutinate with anti-A monospecific serum, and grew in the presence of thionine and fuchsin dyes. Additionally, *Brucella* spp. DNA was detected in tissues from 14 animals (14/59; 23.7%), 12 dolphins (including those with *B. ceti* isolation), 1 *Stenella coeruleoalba*, and 1 *Phoca vitulina* (details on RT-PCR results are presented in the Supplementary File Table S1). The majority of the positive results were attained from the brain, spleen, liver, lung, mesenteric, and pulmonary lymph nodes, uterus, and/or testis. In one suspected animal, DDE040, it was not possible to obtain a pure culture of the isolate; therefore, phenotypic tests were inconclusive. However, the PCR carried out on the DNA extracted from the brain sample showed a positive result. *B. ceti*, isolates were also characterized by the multiplex Bruce-ladder multiplex, and all presented the molecular pattern comprising two fragments of 774 bp and 550 bp. Lastly, MLVA-16 analysis was performed, and data were compared to results obtained by other authors with *B. ceti* strains from different origins, which were deposited in the *Brucella* MLVA database (available at http://mlva.u-psud.fr). In this work, MLVA-11 (panels 1 and 2A markers) discriminated 2 genotypes (GT), dividing *B. ceti* DDE001 into GT8, and DDE002 and DDE003 isolates into GT-17. The results obtained during this study are summarized in Table 1 and in Supplementary File Table S1.

### 3.2. Stranding Data and Necropsy Findings from B. ceti-Positive Animals

Post-mortem investigations were carried out on stranded animals but only included gross findings since histopathological analysis was not available. The necropsy findings described below focus on the observations of the bacteriologically and/or RT-PCR-positive animals. Dolphins DDE001 and DDE002 were juveniles, while DDE003 was an adult. DDE001 was a female dolphin that stranded in March 2022 in Sintra, Portugal (38.8545207, −9.4542235). The animal presented moderate nutritional condition, and a physical exam revealed skin lacerations and traumatic lesions consistent with bycatch. Macroscopic evaluation of internal organs disclosed moderate pulmonary edema and the presence of gas embolism in the renal, mesenteric, and thoracic vasculature. Additionally, mild splenomegaly, with the presence of dark-colored papules (0.5 cm diameter) at the organ’s surface and decreased consistency, was also observed. DDE002 was a male dolphin that stranded in March 2023 in Setúbal, Portugal (38.5094191, −8.9217497). The presence of live ectoparasites suggested a recent death. The animal was in a fair nutritional condition and presented a linear single deep laceration (1.4 cm depth, until the muscular layer) on one side of the body, suggesting a traumatic etiology. Pathological findings included generalized lung congestion and a single ulcerative lesion in the oral cavity (2 cm diameter). DDE003 was a female dolphin that live stranded in July 2023 in Sesimbra, Portugal (38.4887533, −9.1840278). Reports indicate that the animal was disoriented and unable to swim and keep floatable alone. Despite efforts from people present on the beach to refloat the animal, it died shortly after. The animal was severely emaciated. Pathological findings included moderate splenomegaly, generalized lung congestion with associated nematode parasitic infection, the presence of petechiae in the intestinal mucosa, and congested areas in the meninges and the internal side of the skull. Regarding the remaining 11 animals with RT-PCR-positive results (Table 1, Supplementary File Table S1), the causes of death include bycatch, disease, trauma, and some undetermined cases. Bycatch cases frequently showed pulmonary edema, congestion, and lesions to the spleen and liver, such as in DDE008, and DDE013. DDE027 also presented pulmonary congestion and possible parasitism. Disease cases often involved severe pulmonary conditions and parasitism, like in the cases of DDE005 and SCO016, or parasitism alone such as in DDE033. Trauma cases, such as PVI035, showed pulmonary and cardiac congestion. Undetermined cases included DDE036, DDE040 and DDE043. Key findings across all cases highlight frequent pulmonary edema, spleen abnormalities (e.g., hyperplasia, hemorrhage), and parasitism in various organs, indicating common systemic impacts regardless of the cause of death. It is noteworthy to mention that most of these animals exhibited a moderate-to-advanced state of decomposition, which creates significant variability in the interpretation of macroscopic findings.

### 3.3. Comparative Genomic Analysis and Phylogenetic Relationship of B. ceti Isolates

The characterization of the isolates from dolphins DDE001, DDE002, and DDE003 by whole-genome sequencing, at a minimum coverage depth of 200×, confirmed them as members of *B. ceti* species. The key findings are summarized in Table 2.

**Figure 2:**
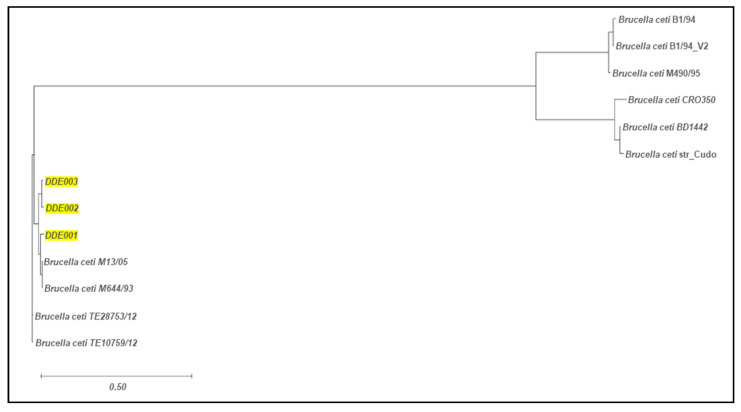
Phylogenetic tree generated by ParSNP. The phylogenetic tree was based on 689 SNVs and is rooted with *Brucella ceti* TE28753-12. The branch length is proportional to the number of SNVs (the scale bar represents the difference in SNVs). The strains from this study are marked with a yellow square.

The phylogenetic relationships of *B. ceti* isolates were assessed using ParSNP analysis, incorporating 10 assembled genomes of *B. ceti* strains available on NCBI as of February 2024 (https://blast.ncbi.nlm.nih.gov/, accessed on 3 April 2024) (see Supplementary Table S2). The resulting phylogenetic tree (Figure 2) highlights the relationships among the Portuguese isolates (DDE001, DDE002, and DDE003) and *B. ceti* strains M13/05/1 and M644/93/1. The phylogenetic reconstruction reveals that DDE002 and DDE003 form a distinct cluster, sharing closer phylogenetic ties to each other, while DDE001 appears to be more closely related to strains *B. ceti* M13/05/1 and M644/93/1. Detailed variations, including SNVs, insertions, and deletions in comparison to the *B. ceti* strains and among the Portuguese isolates, are summarized in Table 2 and Table 3.

**Figure 3:**
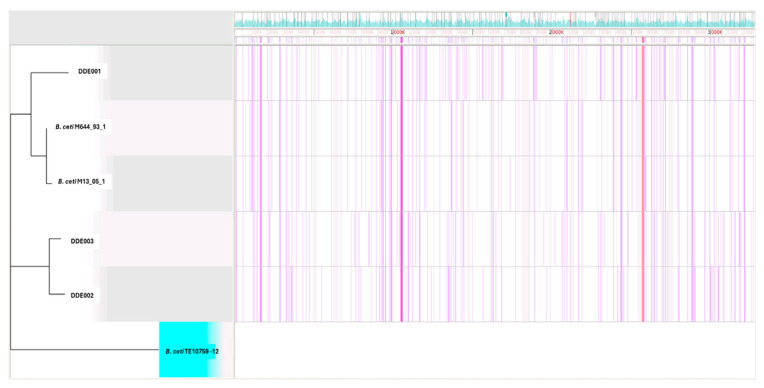
Mutational analysis across *Brucella ceti* isolates DDE001, DDE002, and DDE003, *B. ceti* strain M644_93_1, M13_05_1, and reference strain TE10759-12. The purple lines represent SNVs identified in the isolates, showcasing the distinct mutational patterns. The blue area at the top is the condensed gene annotation density plot from the GenBank file. Image generated with Gingr (v1.3) [26].

Regarding the mutational profile analysis, DDE001 displays a unique pattern of SNVs, while DDE002 and DDE003 exhibit overlapping mutational profiles (Figure 3). DDE001 exhibited 40 specific high-quality SNVs (see Supplementary File Table S3). Key mutations are included in *arg*H (argininosuccinate lyase), involved in arginine biosynthesis (GO:0042450), and in *nh*aA (Na^+^/H^+^ antiporter), which is critical for pH regulation and sodium/proton transport (GO:0006885). Additional mutations were found in genes encoding ABC transporter permeases, implicated in carbohydrate transport (GO:0008643). In contrast, DDE002 and DDE003 shared several mutations. Key shared mutations are included in *odh*B (2-oxoglutarate dehydrogenase), linked to the tricarboxylic acid cycle (GO:0006099), *rsm*G (16S rRNA methyltransferase), involved in ribosomal RNA modification (GO:0046118), and *ruv*B (Holliday junction helicase), essential for DNA recombination and repair (GO:0006310).

VirulenceFinder identified acquired virulence genes in *Brucella* isolates, as detailed in Supplementary File Table S4. For 100% identity and coverage, virulence factors were consistent across all isolates. However, in the core gene *manCcore*, isolates DDE002 and DDE003 showed a 99% identity, indicating a single mismatch when compared to *B. ceti* strain M644/93/1, while DDE001 had a 100% match. The identified virulence genes across the isolates were *bspE*, *vceA*, *btpB*, *manAoAg*, *wbkC*, *virB7*, *lpxK*, *lpxA*, *kds*B, *BPE005*, *acp*XL, *bsp*A, *wzt*, and *manCcore*. The ABRicate pipeline, using CARD and MegaRES databases, identified six acquired antibiotic resistance genes (ARGs): *mprF*, which is commonly linked to resistance to cationic antimicrobial peptides due to its role in modifying cell membrane charge, and genes *bepC*, *D*, *E*, *F,* and *bepG*, generally associated with resistance mechanisms that may include antibiotic modification or efflux (Supplementary File Table S5).

MLST and MLVA profiles (Table 2, and Supplementary File Table S6), revealed that DDE001 aligned with Sequence Type 49 (ST-49) profile (cg-ST392), while isolates DDE002 and DDE003 matched ST-26 profile (cg-ST340). The difference between ST-49 and ST-26 lies at the *cobQ* locus, where a single-nucleotide polymorphism (SNP) occurs at position 213 bp within the 423-bp gene. This SNP is marked by a ‘C’ (allele 6) in ST-49 and a ‘T’ (allele 10) in ST-26. Additionally, MLVA profiling showed that isolate DDE001 exhibited close similarity to *B. ceti* strains M57/07/1 and M260/03/1, which were isolated from the spleen and brain, respectively, of Atlantic white-sided dolphins (*Lagenorhynchus acutus*) in Scotland, with both strains falling under the A1 cluster [32]. Isolate DDE002 was closely related to *B. ceti* strain M654/99/1 from a striped dolphin’s brain, also in Scotland, and classified within the A2 cluster [35]. Meanwhile, DDE003 showed close resemblance to *B. ceti* strains M654/99/1, M83/07/1, M231/07/3, M267/05/4, M83/07/3, and M267/05/1, which were obtained from the brain, kidney, and colorectal lymph nodes of Striped (*Stenella coeruleoalba)*, Atlantic White-Sided, and bottlenose dolphins, all belonging to the A2 cluster [35].

## 4. Discussion

This study investigates *Brucella* infection in marine mammals stranded along the Lisbon and Tagus Valley coast from 2022 to mid-2024. In out of the 59 marine mammals examined, *B. ceti* was isolated in three common dolphins (5.1%). This finding highlight, for the first time, the presence of *Brucella* in marine mammals on Portugal’s coastline, expanding the dispersion area of this bacteria. The observed prevalence (5.1%) is like the 4.92% prevalence obtained by Grattarola and colleagues [10] in cetaceans stranded along the Italian Coastline.

As referred previously, *B. ceti* has been associated with a range of pathological changes in cetaceans. In this study, dolphins in which *B. ceti* isolation was achieved exhibited distinct pathological findings, with some being coincident with the ones expected for *Brucella* infections. For instance, dolphin DDE003, with isolation of *B. ceti* and *Brucella* DNA detection on the central nervous system, presented disorientation and inability to swim and maintain equilibrium and flotation, suggesting a situation of neurobrucellosis, like the findings in a dolphin of the Mediterranean Catalonian coast [4]. However, in the absence of histopathological analysis, a definitive diagnosis of meningitis cannot be made, and further investigation, including histopathological and microbiological analyses, would be required to confirm neurobrucellosis or exclude other potential central nervous system infections. It also displayed severe emaciation, parasitic infection, and multiple signs of systemic distress, including splenomegaly. DDE001 and DDE002 presented pathological changes typically not consistent with *B. ceti* infection, underlying the diverse clinical presentations of *Brucella* infections, already referred by other authors, and complicating the diagnosis in marine mammals. Furthermore, it is interesting to highlight that the three cases display different likely death causes, reinforcing the idea that susceptibility to this pathogen can differ between individuals and be linked to different ecological drivers. This study emphasizes the importance of histopathological and ancillary diagnostic analyses to complement gross findings and improve the understanding of mortality factors in stranded cetaceans. In fact, histopathology is critical for diagnosing conditions such as meningitis, encephalitis, or other organ-specific lesions associated with *Brucella* infections. Consequently, the lack of such data in this study precludes definitive conclusions about the role of *Brucella* in the observed pathological findings, emphasizing the need for future studies to incorporate comprehensive histological examinations to better understand the pathogenic mechanisms and the potential contributions of *Brucella* infections to cetacean morbidity and mortality.

Although *B. ceti* isolation was achieved in 5.1% (3/59) of dolphins, the results obtained by RT-PCR for *Brucella* spp. detection directly from the same tissues were higher, with molecular-positive results in 14 animals (23.7%), including those 3 *Brucella*-positive, suggesting that the incidence of this agent might be higher in these species. Discrepancies between RT-PCR and bacteriological results can occur due to several reasons, including the sample quality, the sensitivity of RT-PCR versus bacteriological culture, stage of the infection, presence of live bacteria, and specificity issues. Bacteriological culture relies on the growth of live organisms, which can be affected by sample storage, the presence of antibiotics, or the viability of bacteria. In fact, most of the RT-PCR-positive animals exhibited a moderate to advanced state of decomposition, which can hamper the isolation of the agent. Also, in early or chronic stages of *Brucella* infection, the bacterial load in tissues may be too low, compromising the culture’s success. On the other hand, depending on the target *locus* and method specificity, RT-PCR is highly sensitive and can detect low levels of *Brucella* DNA, even when viable bacteria are absent or below the limit of detection for culture methods. In fact, the three isolates here identified were obtained in samples from animals with code 1 (recently died) or 2 (fresh carcass) of preservation status [14].

The MLST analysis revealed distinct genetic profiles, indicating diversity within *B. ceti* strains. ST-26 was attributed to the strains from animals DDE002 and DDE003, also previously identified in isolates from dolphins of the Mediterranean Sea and North America. This sequence type is exclusively observed in dolphin isolates, forming cluster A [4,5,8]. The isolate from DDE001 was assigned to ST-49, which was also observed in 17 *B. ceti* isolates, two in Spain and the remaining in Scotland, between 2006 and 2019 (PubMLST, available online: https://pubmlst.org/organisms?title=Brucella+spp. accessed on 15 November 2024). Additionally, MLVA showed a close relation between the isolates from this work and isolates from the Atlantic Sea, although with DDE001 falling under the A1 cluster, and DDE002 and DDE003 falling under the A2 cluster [35]. Up to now, all *B. ceti* Mediterranean strains stem in a separate branch from the main MLVA A1 and A2 clusters of *B. ceti* isolates from dolphins inhabiting the Atlantic Ocean [4,5]. This close MLVA similarity to other dolphin-derived strains supports the hypothesis of specific host-adapted *Brucella* lineages in marine environments [38]. Also, the comparative genomic analysis showed a close relationship with other *B. ceti* strains previously isolated from dolphins, namely those obtained from dolphins in the Scottish Sea [38], indicating possible common sources or transmission pathways within marine environments. In fact, the phylogenetic analysis based on the SNVs revealed that DDE002 and DDE003 form a close cluster (both belonging to cluster A2), suggesting they share a more recent common ancestor. In contrast, DDE001 exhibits a unique position (cluster A1), indicating divergence. Interestingly, DDE001 shows closer phylogenetic ties to the strains M13/05/1 and M644/93/1 than to DDE002 and DDE003, suggesting different selective pressures or environmental adaptations.

The unique SNVs identified in DDE001, particularly in *arg*H and *nha*A, suggest metabolic and transporter-related adaptations. The involvement of *arg*H in arginine biosynthesis and *nha*A in sodium/proton transport points to potential shifts in nitrogen metabolism and osmotic pressure and/or homeostasis, possibly reflecting adaptation to environmental conditions distinct from those encountered by DDE002 and DDE003. Conversely, the shared mutations in DDE002 and DDE003 highlight conserved mechanisms supporting energy production and genome maintenance. Mutations in *odh*B suggest enhanced metabolic functions associated with the tricarboxylic acid cycle, while *rsm*G and *ruv*B underline the importance of ribosomal RNA modification and DNA repair mechanisms, respectively.

Previous studies provide insights into the virulence factors [38,39,40,41,42] and antimicrobial resistance (AMR) genes [39,40,41] present in *B. ceti* isolates, highlighting the importance of monitoring these genes to manage resistance risks in marine mammal pathogens. In the present work, virulence genes were consistent across the three isolates, reinforcing the pathogenic potential of *B. ceti*, with minor variations in the *man*Ccore gene suggesting slight genetic diversity. Thirteen virulence-associated genes were identified: *bspE* gene, associated with pathogenesis, likely aiding in host interaction and enhancing *B. ceti’*s ability to cause disease. While specific studies on *B. ceti* are limited, research on related *Brucella* species suggests that similar genes contribute to host–pathogen interactions [38,39,40,41,42]; *vce*A gene, part of the *virB* operon, is essential for the Type IV secretion system, crucial for the intracellular survival as it allows *Brucella* to manipulate host cells [38,39,40,41]; *btpB* gene is involved in host interactions and the modulation of the host immune response, probably contributing to the ability of *B. ceti* to evade the host immune system [38,39,40,41]; *manAoAg* gene, involved in the biosynthesis of mannose-containing O-antigen [38,39,40,41,43]; *wbk*C gene, associated with O-polysaccharide biosynthesis, which contributes to the structure of the bacterial cell surface [38,39,40,41]; *virB7* gene, also part of the Type IV secretion system, which is crucial for intracellular survival and virulence as this system allows *Brucella* to translocate effector molecules into host cell [38,39,40,41,44]; *lpx*K and *lpx*A genes, involved in lipid A biosynthesis [40,41]; *kds*B, involved in assembling the LPS core by adding KDO (3-deoxy-D-manno-octulosonic acid), ensuring proper LPS structure and contributing to bacterial virulence [40,41,43,44]; *BPE005*, a putative effector protein that may be secreted through the Type IV secretion system, potentially helping *B. ceti* evade host defenses [44]; *acp*XL enhances membrane integrity and resilience against host immune responses [40]; *bsp*A, which is a gene coding for a surface/adhesion protein that likely facilitates bacterial attachment to host cells, aiding colonization and immune evasion [41]; *wzt* is part of the O-antigen transporter (with Wzm), essential for exporting O-polysaccharides and maintaining an intact LPS, which is crucial for full virulence [38,40]; and *manCcore* gene, present in isolate DDE001, required for synthetizing LPS core oligosaccharide, which is vital to *Brucella’*s outer membrane and pathogenicity [40,41,43,44]. Furthermore, the analysis performed using the CARD and MegaRES databases highlighted the presence of six genes potentially involved in AMR in the three *B. ceti* genomes: the multiple peptide resistance factors *mpr*F, and the outer membrane efflux proteins *bepC*, *bep*D, *bep*E, *bep*F, and *bep*G [39,40]. These results were in accordance with other authors [38,39,40,41,42], and raise concerns regarding treatment challenges, highlighting the importance of monitoring ARGs in *B. ceti* to better understand and manage the risks associated with resistance in marine mammal pathogens. These studies collectively reinforce the pathogenic potential of *B. ceti* and the presence of virulence and AMR genes, underscoring the importance of ongoing surveillance and research to effectively manage the risks associated with this marine mammal pathogen.

## 5. Conclusions

In conclusion, the demonstration of the existence of *B. ceti* in dolphins for the first time in Portugal highlights the need for further studies with greater geographic coverage to assess the infection’s prevalence in Portuguese waters. These findings shed light on the occurrence and characteristics of *B. ceti* infection in dolphins, and emphasize the need for continuous surveillance and advanced diagnostic methods to better understand and manage *Brucella* infections in marine mammals. Continued research on *B. ceti* infection is crucial to further understand its transmission dynamics, host range, and its impact on cetacean populations in the Atlantic Sea. Additionally, there is a need for the development of effective diagnostic tools and sustainable management strategies to mitigate the spread of this infection and preserve the health of affected cetaceans.

## Figures and Tables

**Table 1:** Detection and characterization of *Brucella* in stranded Animals: samples and regional Data.

Animal Identification ^1^	Sex/Age ^2^	Preservation Status ^3^	Region	Year of Stranding	RT-PCR-Positive Samples ^4^	Culture-Positive Samples ^4^	*Brucella* Characterization
DDE001	F/J	2	Sintra	2022	B, L, Lg, LNm, S	B, L, Lg, S	*B. ceti*
DDE002	M/J	2	Setúbal	2023	B, L, Lg, LNm, S	L, Lg, LNm, S	*B. ceti*
DDE003	F/A	1	Sesimbra	2023	B, C	B, L, Lg, LNm, S	*B. ceti*
DDE005	F/A	4	Setúbal	2022	B, L, Lg, S	NSC	ND
DDE008	M/A	2	Sesimbra	2022	B, L, Lg, S	NSC	ND
DDE013	M/J	2	Cascais	2022	LNm	NSC	ND
SCO016	M/A	2	Almada	2023	B	NSC	ND
DDE027	M/J	2	Cascais	2023	B, L, Lg, LNm, S, T	NSC	ND
DDE033	F/A	1	Cascais	2023	B, Lg, LNp	NSC	ND
PVI035	M/J	4	Cascais	2023	PS, T	NSC	ND
DDE036	M/A	4	Cascais	2023	LNm	NSC	ND
DDE037	F/A	4	Cascais	2023	LNm	NSC	ND
DDE040	M/J	2	Cascais	2023	B	B	Inconclusive
DDE043	M/J	2	Cascais	2023	PS	NSC	ND

^1^ DDE: *Delphinus delphis*; PVI: *Phoca vitulina*; SCO: *Stenella coeruleoalba.* ^2^ A: adult; F: female; J: juvenil; M: male. ^3^ Carcasses’ preservation status [14]: 1 (just died), 2 (fresh carcass), 3 (moderate decomposition), 4 (advanced decomposition). ^4^ B: brain; C: cervix; L: liver; Lg: lung; LNm: mesenteric lymph nodes; LNp: pulmonary lymph nodes; ND: not done; NSC: no suspected colonies; PS: preputial swab; S: spleen; T: testis.

**Table 2:** Summary of the main genomic results for dolphins *B. ceti* isolates.

*B. ceti* Isolate	*B. ceti* Strain M13/05/1 Alignment	*B. ceti* Strain M644/93/1 Alignment	Virulence Genes and Putative Function	Antibiotic Resistance Gene/Putative Function	MLST	cgMLST	MLVA
**DDE001**	3077 SNVs 279 insertions 257 deletions	5855 SNVs 505 insertions 562 deletions	***bspE***: Surface protein, adhesion ***vceA***: Efflux pump subunit ***btpB***: TIR-domain effector, immune modulation ***manAoAg/wbkC***: O-antigen/LPS biosynthesis ***lpxA*, *lpxK*, *kdsB***: Lipid A/LPS core biosynthesis ***virB7***: Type IV secretion system **BPE005**: Possible effector ***acpXL***: Lipid A modification ***bspA***: Surface/adhesion protein ***wzt***: O-antigen transport ***manCcore*** (only in DDE001): involved in immune evasion and protection against host defenses	***mprF***: Lysyl-tRNA synthetase for membrane modification ***bepC–G***: Efflux proteins or putative efflux regulators	ST-49	cgST-392	Cluster A1
**DDE002**	2956 SNVs 248 insertions 241 deletions	4996 SNVs 439 insertions 457 deletions			ST-26	cgST-340	Cluster A2
**DDE003**	2478 SNVs 212 insertions 215 deletions	4997 SNVs 439 insertions 464 deletions					

MLST: Multi-locus sequence typing (ST: Sequence Type from the typing database https://pubmlst.org/ (accessed on 21 March 2024) for the species *Brucella* spp.); cgMLST: core genome MLST (cgST from the typing database https://pubmlst.org/ for the species *Brucella* spp., accessed on 21 March 2024); MLVA: Multiple-Locus Variable-Number Tandem Repeat Analysis; cluster attribution based on: Maquart et al. 2009 [35].

**Table 3:** Single-Nucleotide Variants (SNVs), insertions, and deletions within the Portuguese isolates.

*B. ceti* Isolates	DDE001	DDE002	DDE003
DDE001		213 SNVs, 12 insertions, 18 deletions	216 SNVs, 16 insertions, 20 deletions
DDE002	213 SNVs, 12 insertions, 18 deletions		107 SNVs, 13 insertions, 11 deletions
DDE003	216 SNVs, 16 insertions, 20 deletions	107 SNVs, 13 insertions, 11 deletions	

## Data Availability

The original contributions presented in this study are included in the article. The WGS data were submitted at NCBI GenBank with the following accession numbers: SAMN45131997, SAMN45131998, and SAMN45131999 for DDE001, DDE002, and DDE003 genome sequences, respectively.

## Supplementary Materials

The following supporting information can be downloaded at: https://www.mdpi.com/article/10.3390/ani15030374/s1, Table S1: Cetacean strandings along the Lisbon and Tagus Valley Coastline (January 2022–June 2024): Animal details, Sample collection, and Bacteriological and Molecular results. Table S2: Assembled *Brucella ceti* contigs from NCBI utilized in ParSNP analysis. Table S3: Multiple sequence alignment and mutational analysis data. Table S4: Identification of acquired virulence genes. Table S5: Identification of acquired antibiotic resistance genes. Table S6: *Brucella ceti* Multi-Locus Sequence Typing (MLST) and Multiple-Locus Variable Number Tandem-Repeat Analysis (MLVA) alleles profiles and clustering.
