# Supplementary material for: Brucella ceti in Common Dolphins (Delphinus delphis) in Portugal—Characterization of First Isolates
Source: Animals (Basel). 2025 Jan 28;15(3):374. doi: 10.3390/ani15030374 (PMC11816080; doi:10.3390/ani15030374)
Supplement: Supplementary file 1 [file animals-15-00374-s001.zip › Supplementary Table S2_Genomes Analysis_R1.pptx]

## Slide 1
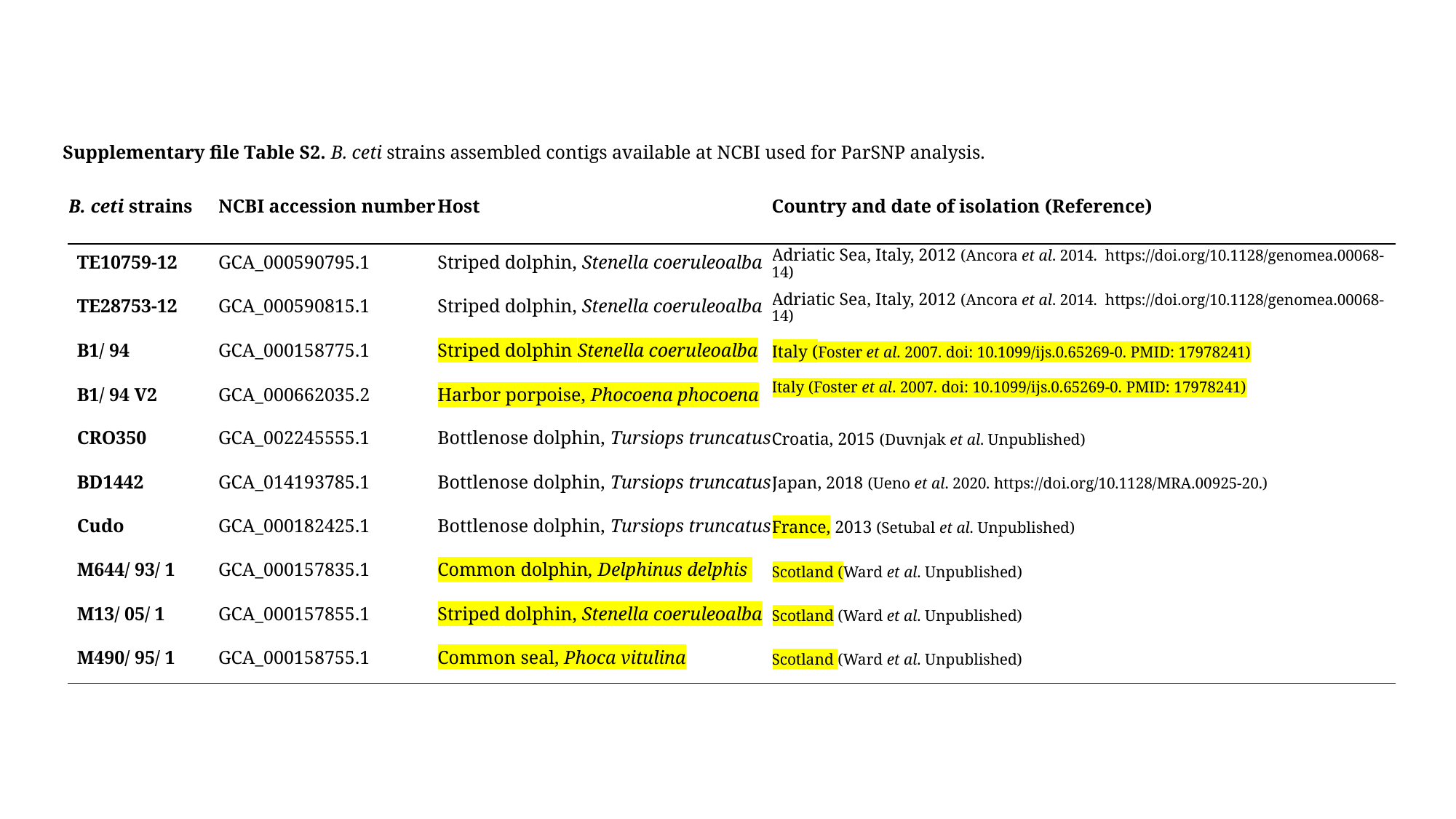

Supplementary file Table S2. B. ceti strains assembled contigs available at NCBI used for ParSNP analysis.
| B. ceti strains | NCBI accession number | Host | Country and date of isolation (Reference) |
| --- | --- | --- | --- |
| TE10759-12 | GCA\_000590795.1 | Striped dolphin, Stenella coeruleoalba | Adriatic Sea, Italy, 2012 (Ancora et al. 2014. https://doi.org/10.1128/genomea.00068-14) |
| TE28753-12 | GCA\_000590815.1 | Striped dolphin, Stenella coeruleoalba | Adriatic Sea, Italy, 2012 (Ancora et al. 2014. https://doi.org/10.1128/genomea.00068-14) |
| B1/ 94 | GCA\_000158775.1 | Striped dolphin Stenella coeruleoalba | Italy (Foster et al. 2007. doi: 10.1099/ijs.0.65269-0. PMID: 17978241) |
| B1/ 94 V2 | GCA\_000662035.2 | Harbor porpoise, Phocoena phocoena | Italy (Foster et al. 2007. doi: 10.1099/ijs.0.65269-0. PMID: 17978241) |
| CRO350 | GCA\_002245555.1 | Bottlenose dolphin, Tursiops truncatus | Croatia, 2015 (Duvnjak et al. Unpublished) |
| BD1442 | GCA\_014193785.1 | Bottlenose dolphin, Tursiops truncatus | Japan, 2018 (Ueno et al. 2020. https://doi.org/10.1128/MRA.00925-20.) |
| Cudo | GCA\_000182425.1 | Bottlenose dolphin, Tursiops truncatus | France, 2013 (Setubal et al. Unpublished) |
| M644/ 93/ 1 | GCA\_000157835.1 | Common dolphin, Delphinus delphis | Scotland (Ward et al. Unpublished) |
| M13/ 05/ 1 | GCA\_000157855.1 | Striped dolphin, Stenella coeruleoalba | Scotland (Ward et al. Unpublished) |
| M490/ 95/ 1 | GCA\_000158755.1 | Common seal, Phoca vitulina | Scotland (Ward et al. Unpublished) |
